# Outcomes of Recurrent Acute Otitis Media in Children Treated for Dental Malocclusion: A Preliminary Report

**DOI:** 10.1155/2016/2473059

**Published:** 2016-11-14

**Authors:** Edoardo Bernkopf, Andrea Lovato, Giulia Bernkopf, Luciano Giacomelli, Giovanni Carlo De Vincentis, Francesco Macrì, Cosimo de Filippis

**Affiliations:** ^1^Dental Clinic, Rome, Italy; ^2^Department of Neuroscience, University of Padova, Audiology Unit at Treviso Hospital, Treviso, Italy; ^3^Dental Clinic, Vicenza, Italy; ^4^Department of Medicine DIMED, University of Padova, Padova, Italy; ^5^Department of Surgery, Otorhinolaryngology Unit, Pediatric Hospital “Bambino Gesù”, Rome, Italy; ^6^Department of Pediatrics and Child Neuropsychiatry, Pediatric Unit, University “La Sapienza”, Rome, Italy

## Abstract

*Aim*. To investigate the role of dental malocclusion treatment in the outcomes of Recurrent Acute Otitis Media (RAOM).* Materials and Methods*. The clinical outcome (number of acute recurrences in 12 months) of 61 consecutive children treated medically for RAOM was analysed. Children underwent an odontostomatologic evaluation, a fiberoptic endoscopy, and skin-prick tests.* Results*. 32 children (group A) were diagnosed with dental malocclusion and treated with a mandibular repositioning plate. Dental malocclusion was ruled out in the other 29 patients with RAOM, and they were used as controls (group B). The two groups were homogeneous in terms of sex, exposure to RAOM risk factors, skin test results, and adenoid hypertrophy, while age was significantly higher in group A. Age, sex, exposure to RAOM risk factors, adenoid hypertrophy, and skin test results were not associated with RAOM outcome. Children in group A treated for dental malocclusion were strongly associated with a lower number of acute episode recurrences at both univariate (*p* < 0.0001) and multivariate analysis (*p* = 0.001).* Conclusions*. RAOM showed better outcomes in children with dental malocclusion wearing a mandibular repositioning device. Dental malocclusion in children with RAOM may play a role in the pathogenesis of Eustachian tube dysfunction.

## 1. Introduction

Otitis media with its different clinical forms (i.e., acute otitis media (AOM), otitis media with effusion, chronic purulent otitis, and chronic otitis with cholesteatoma) has an important incidence in pediatric population. It was estimated that more than 80% of children may experience one or more episodes of AOM in the first three years of life; furthermore, a third of the pediatric population presents recurrent episodes [1, 2]. Recurrent Acute Otitis Media (RAOM) is a clinical form defined as the recurrence of 3 AOM episodes within 6 months or 4 episodes during a year. Environmental (cigarette smoke, exposure to the community) [3, 4] and genetic factors [5] were considered as risk factors for otitis media. Adenoid hypertrophy [6], allergic rhinitis [7], and anatomic anomalies of the nasal pyramid [8] play a role in the pathogenesis of otitis media affecting Eustachian tube function. Medical therapy of acute episodes in RAOM is based mostly on pain medications; antibiotic treatment should be prescribed in severe episodes of AOM [9]. Ventilation tubes should be considered in children with RAOM with middle ear effusion, or in the presence of conductive hearing loss [10].

Epidemiological investigations on dental malocclusion identified a prevalence in children that varied from 20 to 40% [11–13]; it was described to vary among different ethnic groups [11]. To date, there is no evidence-based method of quantification for malocclusion [14]; how deviant occlusal traits should be scored is a matter of serious debate, and this could in part explain the reported variations in malocclusion epidemiology. Common malocclusion pictures are sagittal discrepancies (as the anterior crossbite), vertical discrepancies (as the open bite), or transversal discrepancies (as the deviated bite) [11–13]. The treatment for dental malocclusion is based largely on mandibular repositioning devices [13].

Some structural aspects of the face such as cleft palate [15] and dental malocclusion [16] have been associated with childhood pathologies of the upper airways. Maxillary anatomical alterations have been associated with RAOM, and in such cases, surgical management with adenoidectomy and/or myringotomy with ventilation tube positioning did not ensure a resolution of the disease [17]. De Stefano and colleagues [17] suggested that rapid maxillary expansion could be considered a valid treatment for preventing RAOM in children affected by maxillary anatomical deficits. In adults with otologic complaints, an association was identified with temporomandibular joint (TMJ) disorders [18, 19]. TMJ disorders could influence Eustachian tube patency and middle ear function via a muscle-mediated mechanism [18–20]. In fact, Eustachian tube function/dysfunction has been connected to the functional adequacy of the Tensor Veli Palatini [21] and Levator Veli Palatini muscles [22]. Thus, dental malocclusion affecting TMJ may be involved in Eustachian tube function and in the pathogenesis of RAOM. To date, no previous investigation studied the effect of dental malocclusion treatment on the outcomes of RAOM.

In the present investigation, we included 61 consecutive children treated medically for RAOM. Our hypothesis was that dental malocclusion treatment with mandibular repositioning devices could reduce the number of future episodes of AOM.

## 2. Materials and Methods

### 2.1. Patients

In the present investigation, 61 consecutive children (35 female and 26 male), who were treated medically for RAOM between 2013 and 2014 at the otorhinolaryngology unit of pediatric hospital “Bambino Gesù” (Rome), were included. Informed consent was obtained from the parents of all individuals taking part in the study, and all procedures performed for the purposes of the study complied with the ethical standards of our institutional research committee. The inclusion criteria were as follows: (i) RAOM diagnosis; (ii) no previous ENT surgery. A diagnosis of RAOM was made by the ENT surgeon who enrolled the patients, on the basis of recurrence of 3 AOM episodes within 6 months, or 4 episodes during one year. Previous AOM episodes were clinically documented by the primary care pediatrician.

### 2.2. ENT Evaluation

The primary care pediatrician recommended the first ENT visit as a consequence of recurrent episodes of AOM. All children underwent a complete ENT evaluation in sitting position. The diagnosis of AOM was made following accepted guidelines [9] using signs and symptoms. Tympanometry was performed in doubtful cases. When AOM was diagnosed, amoxicillin was prescribed (40 to 80 mg/kg/day in two doses for 10 days) in severe cases; in nonsevere AOM, the otolaryngologist could either prescribe antibiotic treatment or offer observation with close follow-up based on joint decision-making with the parents [9].

During the first evaluation, fiber optic endoscopy was also performed to assess adenoid tissue: adenoids were graded from 1 to 4 according to the increasing rhinopharyngeal obstruction [23].

### 2.3. Allergological Evaluation

All children underwent skin-prick tests, according to internationally accepted procedures [24]. We used a panel made of 18 diagnostic extracts (Lofarma SPA, Milan, Italy): hazel, alder, birch, plane, cypress, grass mix, olive, mugwort, ragweed,* Alternaria alternata*,* Cladosporium herbarum*,* Aspergillus fumigatus*,* Parietaria*, cat, dog,* Dermatophagoides pteronyssinus*,* Dermatophagoides farinae*, and cockroach.

### 2.4. Odontostomatological Evaluation

All cases underwent the following examination: (i) alginate dental impressions and development in plaster; (ii) pictures of the face with front and profile shots; (iii) pictures of the dental arches. The analysis of the malocclusion was performed in three dimensions; children were diagnosed with sagittal discrepancies (increased overjet or anterior crossbite), vertical discrepancies (open bite or deep bite), or transversal discrepancies (deviated bite or unilateral crossbite). Patients were divided into two groups according to the result of the odontostomatological evaluation: group A, children with a diagnosis of dental malocclusion; and group B, children without malocclusion.

### 2.5. Treatment

**Figure 1:**
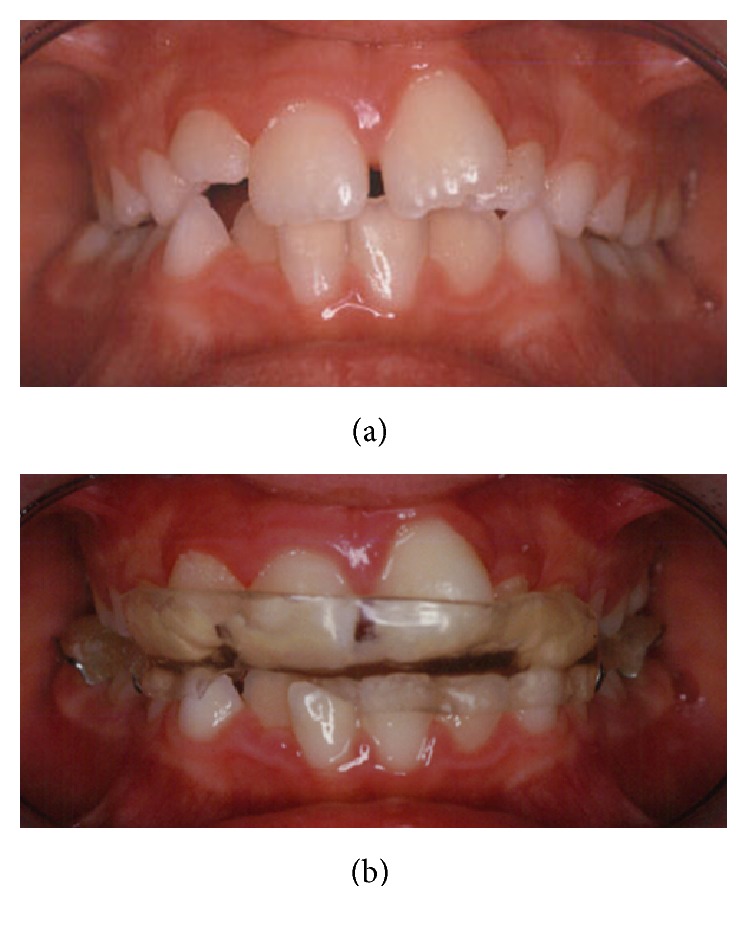
(a) A right deviated bite. (b) The same children wearing the mandibular repositioning device.

Dental malocclusion (group A) was treated with a mandibular repositioning plate (Bernkopf-Bertarini device) [25] adapted for children (Figure 1). Every device was produced by an orthodontic laboratory on a wax bite template that had been made by the orthodontist based on the best patient occlusion. Children chose the device color in order to increase their compliance to treatment. The mandibular repositioning device was kept in place continuously, with the only exception of main meals.

Both groups underwent a topical medical therapy with mometasone furoate nasal spray (50 mcg/nostril twice a day).

### 2.6. Follow-Up Period

The follow-up period was 12 months. The orthodontist evaluated the patients monthly for their tolerance to treatment and modified, if necessary, the oral device to allow orthodontic movements of teeth.

ENT follow-up visits were scheduled at 3, 6, and 12 months. Parents were instructed to contact the ENT service of pediatric hospital “Bambino Gesù” when children complained otalgia or experienced fever, in order to arrange close additional evaluations. Thanks to the extra ENT visits, all AOM episodes were diagnosed by an otolaryngologist. Nasal therapy was continued in children with AOM recurrences. Asymptomatic children were told to stop nasal therapy.

### 2.7. Outcomes Measure

The number of AOM recurrences in 12 months of follow-up was considered the primary outcome.

### 2.8. Statistics

Given the purposes of the investigation, we conducted one intergroup (group A versus group B) analysis and another analysis to detect potential associations between the considered variables and RAOM outcomes in terms of number of AOM recurrences during the 12 months of follow-up.

We decided to dichotomize the considered variables, as we have previously reported [26, 27]: age ≤6 years (age median value) versus >6 years; negative skin-prick test versus positive test; absence of adenoid hypertrophy (grade 1) versus presence of hypertrophy (grades 2–4); number of AOM recurrences 0 versus ≥1. The *t*-test corrected for unequal variance and Fisher's exact test were used when appropriate. Odds ratio (OR) and 95% confidence interval (CI) were calculated. A *p* value <0.05 was considered statistically significant.

A multivariate logistic regression was applied, adding the considered variables with a *p* value ≤0.40 disclosed by Fisher's exact test to the model. The *p* values were calculated with the Wald test. During the analysis, the model was checked for multicollinearity with a variance inflated factor test.

The STATA™ 12.0 (Stata Corp., College Station, TX, USA) statistical package was used for all analyses.

## 3. Results

### 3.1. Intergroup Analysis

Odontostomatological evaluation identified a malocclusion in 32 patients (group A). Dental malocclusion in group A was classified as follows: sagittal discrepancies (increased overjet or anterior crossbite) in 15 cases, vertical discrepancies (open bite or deep bite) in 10 cases, and transversal discrepancies (deviated bite or unilateral crossbite) in 7 cases. The other 29 children (group B) showed a normal occlusion.  Table 1 lists the experimental group characteristics.

The mean age of children in group A was significantly higher than the ones in group B (*t*-test corrected for unequal variance; *p* = 0.013). Fisher's exact test ruled out any significant differences in the distribution of sex (*p* = 0.62), skin-prick tests positivity (*p* = 0.23), or adenoid hypertrophy (*p* = 0.79) between the two groups. There were no differences in terms of RAOM risk factors (passive smoke exposure or day-care/school attendance) between groups A and B.

In the year before the inclusion in the study, the mean number of AOM episodes that occurred in 12 months was slightly higher in group A (6.3 episodes, standard deviation [SD] 2.7 episodes) than in group B (6.0 episodes, SD 2.4 episodes), but not significantly (*t*-test corrected for unequal variance; *p* = 0.61). Children were enrolled continuously in every season, even if the majority of them in both groups were included during autumn and winter (24 children in group A and 22 in group B).

### 3.2. RAOM Outcomes

All children completed the follow-up. The mean cumulative duration of middle ear effusion was 3.6 months (SD 1.9 months) in group A and 6.8 months (SD 2.3 months) in group B. In the 12 months of follow-up, the mean number of AOM recurrences was 0.4 (SD 0.4 episodes) in group A and 5.0 (SD 2.7) in group B. Twenty-nine children in group A did not experience any recurrence of AOM episodes, compared to only 6 patients in group B. Children in group A treated for dental malocclusion had a significantly lower number of AOM recurrences during follow-up (Fisher's exact test, *p* < 0.0001; OR = 37, 95% CI 7.18–233.0). Fisher's exact test did not find significant differences in the distribution of RAOM outcomes when children were stratified according to age (*p* = 0.40), sex (*p* = 0.72), skin-prick test results (*p* = 0.25), adenoid hypertrophy (*p* = 1.0), or passive smoke exposure (*p* = 1.0).

Multivariate estimates of AOM recurrences were based on a logistic model and on the assumption that there were no correlations between significant variables in the final model. No multicollinearity between variables was detected by variance inflated factor. As mentioned previously, the variable selection procedure using backward elimination was set at *p* > 0.40 for Fisher's exact test. The model obtained demonstrated that children in group A treated for dental malocclusion showed a positive prognostic significance in relation to the number of AOM recurrences (Wald test, *p* = 0.001). Age (Wald test, *p* = 0.56) and skin-prick test results (Wald test, *p* = 0.45) were not prognostically significant in relation to the number of AOM recurrences.

The duration of nasal treatment was significantly longer in group B (4.7 months, SD 2.7 months) than group A (3.2 months, SD 1.9 months) (*t*-test corrected for unequal variance; *p* = 0.012). None of the children treated with mandibular repositioning devices reported intolerance to the treatment during follow-up. In all cases, the oral device needed one to three modifications during the follow-up, to allow orthodontic movements of the teeth and maintain the correct occlusion. At the end of the follow-up period of the present study, the orthodontist addressed children with positive outcomes to fixed dental braces, in order to consolidate the correct occlusal state.

## 4. Discussion

According to the results of the present investigation, RAOM showed a significant better resolution in children treated for dental malocclusion (group A) than in children with normal occlusion (group B). All but three patients in group A experienced no AOM recurrences, compared to only 6 in group B, supporting our hypothesis that dental malocclusion treatment with a mandibular repositioning device reduces the number of AOM recurrences.

The main weakness of the present study concerned the type of included patients in the control group: we used children with RAOM for whom dental malocclusion was ruled out. It was questionable if these patients could really be controls for the experimental group: the ideal control cohort would have included children with RAOM and dental malocclusion not wearing the oral device. Unfortunately, we did not find any parents willing to participate in such an investigation; in our experience, when children are diagnosed with dental malocclusion, parents usually demand treatment. Furthermore, since patients in control group had normal occlusion, we could have expected those subjects to have less RAOM episodes, yet the difference was not significant between the groups.

After 12 months of follow-up, 9.4% of children in group A and 79.3% in group B experienced recurrence of AOM episodes. The two groups were homogenous in terms of male to female ratio, RAOM risk factors, skin test results, and adenoid hypertrophy, while age was significantly higher in group A. Patients in group A, treated for dental malocclusion, had a significantly lower number of AOM recurrences in both univariate (*p* < 0.0001) and multivariate analysis (*p* = 0.001). Statistical analysis ruled out any associations between adenoid hypertrophy or skin-prick test results and the number of AOM recurrences. Interestingly, age, the only different characteristic between the two groups, was not associated with the outcome in either univariate or multivariate analysis.

According to recent guidelines, ventilation tubes should be considered in children with RAOM [10]. In Gebhart randomized controlled trial (RCT) on 108 children, patients treated with ventilation tubes experienced 1.3 fewer AOM episodes over a period of six months [28]. Another RCT study included 44 children and randomized the children's ears [29]. All children had ventilation tubes inserted in one ear and either paracentesis or no treatment on the other ear: ear with ventilation tubes resulted in 1.6 fewer episodes of AOM in 12 months of follow-up [29]. A recent investigation on a large cohort of 300 children reported 0.55 fewer episodes of AOM over 12 months in children with ventilation tubes [30]. In the present investigation, children treated for malocclusion had a mean reduction of 5.9 AOM episodes in 12 months of follow-up, while patients treated medically had 1.0 episodes less in average. Remarkably, 90% of patients treated for malocclusions experienced no AOM recurrences. However, ventilation tubes are usually offered to young children: Kujala et al. [30] operated on children aged from 10 months to 2 years. On the contrary, mandibular repositioning devices are usually offered to older children. Also, in this study, we treated with the oral devices patients aged from 3 to 11 years, with a median age value of 6 years.

According to our results, RAOM showed a significantly better resolution in children wearing a mandibular repositioning device for dental malocclusion than in the group for which dental malocclusion was ruled out. Dental malocclusion, in this population, may play a role in RAOM pathogenesis with a muscle-mediated mechanism. In fact, Eustachian tube function/dysfunction has been connected to Tensor Veli Palatini [21] and Levator Veli Palatini muscles [22] functionality, which could be altered in patients with cranial skeletal development syndrome. Another possible mechanism could involve a TMJ disorder that influences Eustachian tube patency [18–20]. Normally, mandibular condyles are located next to the anterosuperior wall of the joint cavity, but, in children with disharmonic craniofacial development, the condyles could dislocate to a rear position in the joint cavities, closer to Eustachian tube and middle ear.

## 5. Conclusions

According to our results, dental malocclusion in children with RAOM might play a role in the pathogenesis of Eustachian tube dysfunction. In our case series, RAOM showed better outcomes in children with dental malocclusion wearing a mandibular repositioning device. Children from the age of three would benefit more from this device. Otolaryngologists should look for dental malocclusion in children with middle ear diseases, with the possibility of referring them for orthodontic care.

## Figures and Tables

**Table 1:** Patients' characteristics according to group distribution (group A: dental malocclusion, group B: normal occlusion).

	Group A	Group B
Number of patients	32	29
Female/male patients	20/12	15/14
Mean age in years (SD^*∗*^)	6.6 (1.9)	5.3 (1.9)
Patients with positive skin test	3	0
Patients with adenoid hypertrophy	13^†^	10^‡^
Patients exposed to passive smoke	3	3
Day-care or school attendance (%)	100	100
Number of previous acute episodes in 12 months (SD)	6.3 (2.7)	6.0 (2.4)

^*∗*^SD: standard deviation.

^†^Grade 2 in 8 patients and grade 3 in 5.

^‡^Grade 2 in 6 patients and grade 3 in 4.
